# Consistency in bacterial extracellular vesicle production: key to their application in human health

**DOI:** 10.20517/evcna.2024.76

**Published:** 2025-01-17

**Authors:** Ke Dai, Bo Liao, Xiaotian Huang, Qiong Liu

**Affiliations:** ^1^Department of Medical Microbiology, School of Basic Medical Sciences, Jiangxi Medical College, Nanchang University, Nanchang 330006, Jiangxi, China.; ^2^First Clinical Medical College, Jiangxi Medical College, Nanchang University, Nanchang 330006, Jiangxi, China.; ^#^Authors contributed equally.

**Keywords:** Bacterial extracellular vesicles, heterogeneity, purification, application

## Abstract

Bacterial extracellular vesicles (BEVs) are naturally occurring functional structures that play critical roles in bacterial life processes. These vesicles, commonly known as outer membrane vesicles (OMVs), were first found to be released by Gram-negative bacteria; however, it has since been confirmed that Gram-positive bacteria also secrete BEVs. As research advances, BEVs are increasingly utilized in diverse applications, including vaccine development and drug delivery. Nevertheless, the effective employment of BEVs in these contexts requires the acquisition of vesicles with consistent properties and functions through appropriate culture, isolation, and purification methods. This review examines the advantages and disadvantages of various purification techniques alongside the heterogeneity they may introduce. We utilize the heterogeneity of BEVs as a framework to critically analyze the barriers to their application and the factors influencing their characteristics. Additionally, we constructively propose solutions to enhance the consistency of BEVs, thereby facilitating their further development and application.

## INTRODUCTION

BEVs are composed of lipid bilayers derived from bacterial membranes, typically ranging from 20 to 500 nm in diameter^[1]^. Bacterial extracellular vesicles (BEVs) include vesicle-like structures produced by both Gram-positive and Gram-negative bacteria^[2]^. The latter, often referred to as outer membrane vesicles (OMVs), originate specifically from the outer membrane of Gram-negative bacteria^[3]^. Proteomic and biochemical analyses reveal that the membrane structure of these vesicles incorporates structural proteins, phospholipids (PLs), and lipopolysaccharides (LPS), while their inner lumen contains various substances sourced from the periplasm or cytoplasm of the parent bacterium, including cytoplasmic proteins and nucleic acids^[4]^. Due to their distinctive size and composition, BEVs can traverse distances inaccessible to the bacteria themselves, facilitating the release of bioactive substances and fulfilling diverse biological functions. Notably, BEVs play significant roles in substance delivery and immune modulation. They have potential applications as immunomodulators in therapies for inflammatory diseases and tumors, as well as in vaccine development to reduce infection rates and morbidity^[5,6]^.

Despite the vast potential applications of BEVs, the current transition of BEVs from laboratory research to industrial production and clinical application remains significantly constrained. The primary challenge lies in the heterogeneity of BEVs. This heterogeneity refers to the variability in structure, composition, size, and function of BEVs, which can arise from differences in bacterial strains, growth conditions, and environmental factors. Such heterogeneity often reflects the functional or stress states of the cells and results from complex regulatory mechanisms and adaptive changes necessary for maintaining intracellular homeostasis. Moreover, various culture conditions, including temperature and duration, along with the chosen purification methods, contribute substantially to this heterogeneity. These factors can compromise the structural integrity and final yield of BEVs, potentially leading to aggregation and alterations in composition, which may adversely affect subsequent proteomic analyses and sample homogeneity^[7,8]^. Therefore, it is crucial to address the heterogeneity of BEVs by thoroughly analyzing the influencing factors and purification methods to obtain vesicles with consistent yields and properties for future studies and applications.

## METHODS OF ISOLATION OF BEVS

The isolation of BEVs involves extracting and purifying vesicles from bacterial cultures to obtain a pure sample for subsequent studies. Most protocols for BEV isolation begin with one or more low-speed centrifugation steps, followed by filtration of the supernatant to produce a cell-free filtrate. The resulting filtrate is then further processed, concentrated, and purified by a range of techniques^[9]^. Various methods are available for purifying BEVs, including ultrafiltration, ultracentrifugation, density gradient centrifugation, immunoaffinity, microfluidics, or combination thereof. Each of these purification methods has its own advantages and disadvantages. Analyzing and clarifying these purification techniques is essential, as it could lead to the development of improved methods aimed at enhancing the purification efficiency of BEVs.

### Ultrafiltration

Ultrafiltration employs membrane filters with varying pore sizes to separate particles or vesicles of specific sizes and is often used as a complementary method in BEV separations. Notably, ultrafiltration can serve as a replacement for continuous ultracentrifugation stages^[10]^. However, clogging of membrane pores can decrease separation efficiency, increase contaminant concentrations, and lead to the loss of target BEVs. Utilizing membranes made from hydrophilic materials with low affinity for proteins, such as hydrophilized polyvinylidene difluoride membranes, helps minimize the irreversible adsorption of BEVs, thereby reducing losses^[11]^. In comparison, ultracentrifugation is a time-consuming process that often results in variable recovery rates due to differences in rotor types used by researchers. Additionally, repeated ultracentrifugation steps may damage isolated vesicles, compromising their quality. Ultrafiltration, in contrast to ultracentrifugation methods, offers higher particle yields and separation efficiencies, and significantly reduces processing time. This method is particularly well-suited for producing clinical-grade BEVs compared to traditional ultracentrifugation protocols^[12]^. However, it is important to note that the choice of ultrafiltration membrane pore size can impact separation outcomes. This variability may lead to differences in protocols across laboratories, complicating result comparisons. Furthermore, ultrafiltration is not effective at removing non-BEV protein contaminants from biological fluids, which can affect analyses of BEV protein content. Therefore, further optimization is necessary to enhance the consistency and reproducibility of the separation process.

### Ultracentrifugation

Ultracentrifugation operates on the principle that particles with different densities will pellet at varying g-forces under high-speed centrifugal force, enabling the separation of different types of extracellular particles, such as large extracellular vesicles (lEVs), small extracellular vesicles (sEVs), exomeres, and supermeres^[13]^. Typically, depending on the growth curve (6-60 h), the production of BEVs in a laboratory or industrial setting begins with a longer incubation time in liquid media. Upon completion of the culture, recovery of BEVs involves low-speed centrifugation and filtration to remove intact bacterial cells as well as any other insoluble and unwanted debris. Sterile filtration was then performed in 0.22 and 0.45 μm filters, depending on the size of the bacteria and vesicles. However, the use of 0.22 μm filters may reduce yield by retaining larger vesicles (200-400 nm). Additionally, ultrafiltration is often used to supplement this. It takes the cell-free supernatant obtained after multiple low-speed centrifugation filtrations and ultrafilters it using a 100-500 kDa ultrafiltration membrane to remove uncoated proteins. Finally, ultracentrifugation is used to obtain the BEV-enriched fraction. The obtained BEV pellet was resuspended in PBS and stored at -20 or -80 °C^[14,15]^. During this operation, the selection of centrifugal forces requires a careful balance between separation efficiency and purity. Higher centrifugal forces (e.g., 167,000 g) can efficiently isolate sEVs but may also result in the co-isolation of non-vesicular particles (NVs). Conversely, lower centrifugal forces (e.g., 120,000 g) may reduce the co-isolation of NVs but might not completely isolate all sEVs^[13]^. This method is simple to execute and relatively less technically demanding, making it one of the most accessible methods for BEV isolation. However, some contaminants such as hyphae, flagella, and soluble components are still present. They cannot be separated by centrifugation and are still present in the BEVs precipitate. Therefore, additional separation steps, such as density gradient centrifugation, are usually required to produce high-purity BEVs. Ultracentrifugation requires repeated centrifugation or additional preconcentration steps if large sample volumes are processed, so it is hardly suitable for large-scale production of BEVs^[9]^. In addition, ultracentrifugation can lead to BEV aggregation, which may produce artifacts during flow cytometry and single-particle tracking (SPT) analyses^[16]^. Moreover, considerations such as the cost of high-speed ultracentrifuges, as well as the time and labor involved in processing samples, must be taken into account. Under ultracentrifugation, both BEVs and NVs may become heterogeneous, resulting in the generation of distinct subpopulations. Some soluble proteins and nucleic acids may also be co-isolated with each fraction^[13]^. This implies that the vesicles obtained through this purification method may lack stability and exhibit issues with reproducibility and consistency.

### Density gradient centrifugation

Density gradient centrifugation is a widely used biological separation technique that capitalizes on the different settling velocities of biomolecules within a density gradient, facilitating the separation and purification of various components within a biological sample^[17]^. BEVs are less dense than soluble secretory proteins, flagella, and cilia due to their lipid composition. Consequently, during density gradient centrifugation, BEVs migrate to lighter fractions, completing their purification. This method offers several advantages, including effective separation, high precision, and broad applicability. The preparation of density-purified vesicles is particularly recommended for studying vesicles that contain various factors from bacteria, such as *Pseudomonas aeruginosa*^[18]^.

In density gradient centrifugation, samples are subjected to a gradient created by adding the sample to a density gradient medium. Components of the sample with differing densities will settle at various locations during centrifugation, resulting in stratification. Thus, the choice of density gradient medium is crucial for the efficient isolation and purification of BEVs. The most commonly utilized density gradient medium is iodine glycol, which, at effective concentrations, is isotonic. This isotonicity helps preserve fine membrane structures, suggesting that iodine glycol can enhance the concentration of purified BEVs compared to other media. Furthermore, employing density gradient centrifugation in the BEV purification process serves as an effective strategy to improve the consistency of purified BEVs, especially after enrichment via ultracentrifugation^[19]^.

### Immunoaffinity

BEV isolation based on immunoaffinity capture is considered a state-of-the-art method for purifying specific classes of BEVs. The technique relies on the use of antibodies to capture BEVs with specific protein markers on their surfaces. Tetratransmembrane proteins, such as CD81 or CD63, are ideal immunocapture proteins because they are enriched on vesicle membranes^[20]^. Immunoaffinity capture can be achieved by incubating samples with magnetic beads coated with antibodies against surface proteins or gold-loaded iron oxide nanocubes for the isolation of BEVs. There are other affinity methods that use markers from parental cells, such as chondroitin sulfate peptidoglycan4, epithelial cell adhesion molecules (EPCAM), or exosome binding molecules such as heat shock proteins and heparin^[21]^. Immunoaffinity methods for marker-specific isolation or removal of BEVs can then be combined with STED microscopy to visualize individual BEVs and their surface marker profiles to gain insight into BEV heterogeneity. Based on the protein profiles of BEVs isolated by immunoaffinity, this method may be “the most efficient way to isolate exosomes” compared to ultracentrifugation and gradient density separation^[22]^. Immunoaffinity, as a technique for targeted isolation of BEVs, has some advantages in terms of short time and high purity, but it also has significant drawbacks. First, this method is costly because specific antibodies or affinity agents are required and the procurement and experimental processes are costly. Second, the yield of the immunoaffinity method is generally low, which may not meet the needs of large-scale research or clinical applications. In addition, the method is highly dependent on knowledge of specific surface markers, and the selection of suitable targets will become difficult without sufficient biological information. Finally, the process of removing exosomes from antibodies or affinity agents may mask the selection of the actual target or the molecules required for action, affecting the accuracy and reliability of the study results^[23]^. Therefore, despite the importance of immunoaffinity methods in biomedical research, their limitations need to be fully appreciated in order to promote the development of more effective separation techniques.

### Microfluidics

As an emerging technology, microfluidics provides an effective platform for the separation of BEVs. This is because it enables precise control of particle physical properties under well-defined fluidic conditions and integration of multiple processes into a single system. These advantages are expected to improve separation performance, simplify operational procedures, and reduce the risk of sample loss and cross-contamination. As a result, this technology has been adopted for implementing standard assays as well as exploring new principles for rapid separation and analysis of clinical-grade BEVs for diagnostic and therapeutic applications^[24]^. Microfluidic systems use either labeled or label-free methods. Labeling-based methods use immunoaffinity interactions to specifically separate vesicles from a mixture of other components. Examples include the use of microfluidic devices such as the ExoChip to improve the specificity of exosome separation. The device has a capture efficiency of 38% for healthy exosomes and 90% for cancer cell exosomes. In contrast, label-free methods use and isolate vesicles based on physicochemical differences such as size and density. For example, different acoustic forces are applied to BEVs based on their size and density, and particles of different sizes are isolated directly from the sample. This approach has the advantage of preserving the nature, structure, and function of the BEVs, shortening processing time, facilitating integration, and reducing human intervention^[25]^. Overall, microfluidics overcomes the limitations of traditional methods by allowing the use of small portions of raw samples to perform a variety of routine laboratory operations in a short period of time to meet the needs of immediate clinical testing. In addition, it has a high throughput capability, i.e., a unique potential for large-scale bioassays. This method enables more efficient recovery and isolation of specific subtypes of BEVs while reducing non-vesicular co-isolates, thereby reducing background interference in subsequent biomarker analysis steps. However, there are still points that deserve our attention in practical applications. Microfluidic devices vary widely in BEV separation performance. In addition to maximizing throughput, recovery, and purity, other factors such as capacity, sample volume, enrichment, co-separates, and the potential for clogging must be considered. There are also issues with labeling-based techniques requiring expensive antibodies, and many microfluidic techniques requiring complex chip fabrication, among others^[26,27]^.

### Differences in the purification of BEVs from Gram-negative and Gram-positive bacteria

The Gram-negative bacterial cell wall is thin and consists of two membrane layers separated by a periplasm, which has an outer membrane structure. In contrast, Gram-positive bacterial cell walls have a unique membrane structure consisting of a membrane and a thick peptidoglycan layer^[28]^. Such structural differences affect the biogenesis and purification processes of BEVs. For example, in Gram-negative bacteria, there are three main hypotheses for vesicle formation: (1) membrane curvature-inducing proteins induce the formation of spherical vesicles by increasing membrane curvature; (2) the cross-linking dissociation between the outer membrane and the peptidoglycan facilitates vesicle production; and (3) mutations in the VacJ/Yrb ATP-binding cassette (ABC) transport system trigger vesicle production. In contrast, the biogenesis of BEVs in Gram-positive bacteria has been challenging due to their peptidoglycan-rich structure. It is now believed that vesicle formation in Gram-positive bacteria is primarily mediated by the growth of specific lipid-rich regions within the cytoplasmic membrane, which subsequently weakens the thick peptidoglycan layer in the presence of endolysin and facilitates vesicle release^[29]^. Therefore, there are some differences in the methods and steps used in the isolation of BEVs between Gram-positive and negative bacteria, due to differences in cell walls and secretion mechanisms.

For gram-positive bacteria, ultracentrifugation and washing are commonly employed, with additional purification techniques sometimes added to improve the purity of BEVs. Physical and chemical treatments of the outer membrane are usually not required. For example, in the preparation of BEVs from representative Gram-positive bacterial strains such as *Lactobacillus*^[30-32]^, *Streptococcus pneumoniae*^[33]^, and *Staphylococcus aureus*^[34]^, ultracentrifugation is typically employed to isolate the vesicles, and the resulting precipitate is then washed with PBS or other suitable buffers to remove impurities. For further purification of crude BEVs from the co-isolates, either size exclusion chromatography or OptiPrep (iodixanol) density gradient medium method is commonly utilized. There are also studies mentioning the importance of washing. In contrast to the Gram-negative *Klebsiella pneumoniae* MV, further washing steps may be required for *Streptococcus pneumoniae* (*S. pneumoniae*) MV. Because of the lower density of the latter, even small amounts of OptiPrep™ residue are difficult to settle and, therefore, challenging to wash. After the first aqueous wash, the pellet may not be clearly visible, appearing instead as a cloudy residue floating at the bottom of the test tube. If this occurs, the liquid fraction needs to be carefully collected until 1-2 mL of liquid remains at the bottom, and the water wash repeated until the OptiPrep™ is completely cleaned and visible particles have formed (3 repeated washes are required)^[35]^. The isolation and purification process of Gram-negative BEVs is even more complex, considering that the outer membrane is enriched with LPS and other components. For example, OMV is chemically extracted from whole bacteria using a decontaminating agent (e.g., deoxycholate), which produces vesicular aggregates of insoluble outer membrane proteins called detergent-extracted OMV (dOMV). The fermentation supernatant containing spontaneous vesicles is usually discarded and OMV extraction is then performed. A sonication step can be introduced to obtain smaller, more homogeneous dOMV, or sterile filtration can be simplified. The use of decontaminants can greatly reduce LPS and lipoprotein content, thereby reducing reactogenicity and improving OMV tolerance^[36]^.

Despite these differences between Gram-positive and Gram-negative bacteria, the variability in their EV purification and isolation is not currently clearly distinguished in many studies. For example, Watson *et al*. describe a scalable workflow for BEV isolation, which involves centrifugation, filtration, ultrafiltration, and size-exclusion chromatography. This protocol is applicable to other human gut-associated Gram-negative and Gram-positive species^[37]^. However, it is important to note that when purifying BEVs from Gram-negative and Gram-positive bacteria using the same method, certain parameters - such as centrifugation force, centrifugation time, and the setting of the density gradient - must be adjusted depending on the bacterial strain^[35]^. Additionally, Liu *et al*. summarized a set of isolation methods suitable for most bacteria, including Gram-negative bacteria (e.g. *Escherichia coli* Nissle 1917 and *Akkermansia muciniphilia*) and Gram-positive bacteria (e.g. *Lactobacillus rhamnosus* GG). In their protocol, bacteria and their debris were first completely removed from the fermentation broth by low-speed centrifugation (1,000-2,000 g) and 0.22 μm sterile filters. Then, non-BEV-related proteins were eliminated using 100 kDa ultrafiltration membrane. Finally, BEVs were isolated and purified by ultracentrifugation (100,000 g) and iodixanol gradient centrifugation^[38]^.

It is also worth noting that in a newly published paper on the production of *Lactobacillus* BEVs, it is mentioned that although previous studies have focused on optimizing isolation strategies, the impact of pre-isolation factors on BEV production and purity cannot be ignored. The researchers systematically varied three key process parameters, including medium pH, growth time, and broth concentration of the probiotics, and found that they affected critical quality attributes (CQAs), including physical properties and biological composition^[39]^. Although it is not yet clear whether these effects are consistent across other Gram-positive and Gram-negative bacteria, the findings suggest that attention should be given to the culture process in addition to isolation and purification.

### Heterogeneity of BEVs

A review of various purification methods indicates that the choice of techniques and strategies significantly influences the quantity, quality, and function of BEVs produced. BEVs exhibit heterogeneity in terms of size, shape, and composition, which arises from their production by various cell types or under different cell states. This heterogeneity complicates the purification processes, presenting a major limitation to the large-scale application of BEVs. Besides the purification strategy itself, numerous factors can further contribute to this heterogeneity, making it more difficult to standardize BEV production. As researchers increasingly recognize the importance of consistent BEVs for clinical and laboratory applications, the issue of heterogeneity has become a key focus. Variability in BEVs affects vesicle purity, yield, and scalability, severely hampering their advancement in biotechnology and restricting their clinical use^[15]^. In the following sections, we will review potential and specific examples of heterogeneity in BEV preparations, and discuss the implications of such heterogeneity for practical applications of BEVs.

## APPLICATION BARRIERS OF BEVS DUE TO HETEROGENEITY

**Figure 1:**
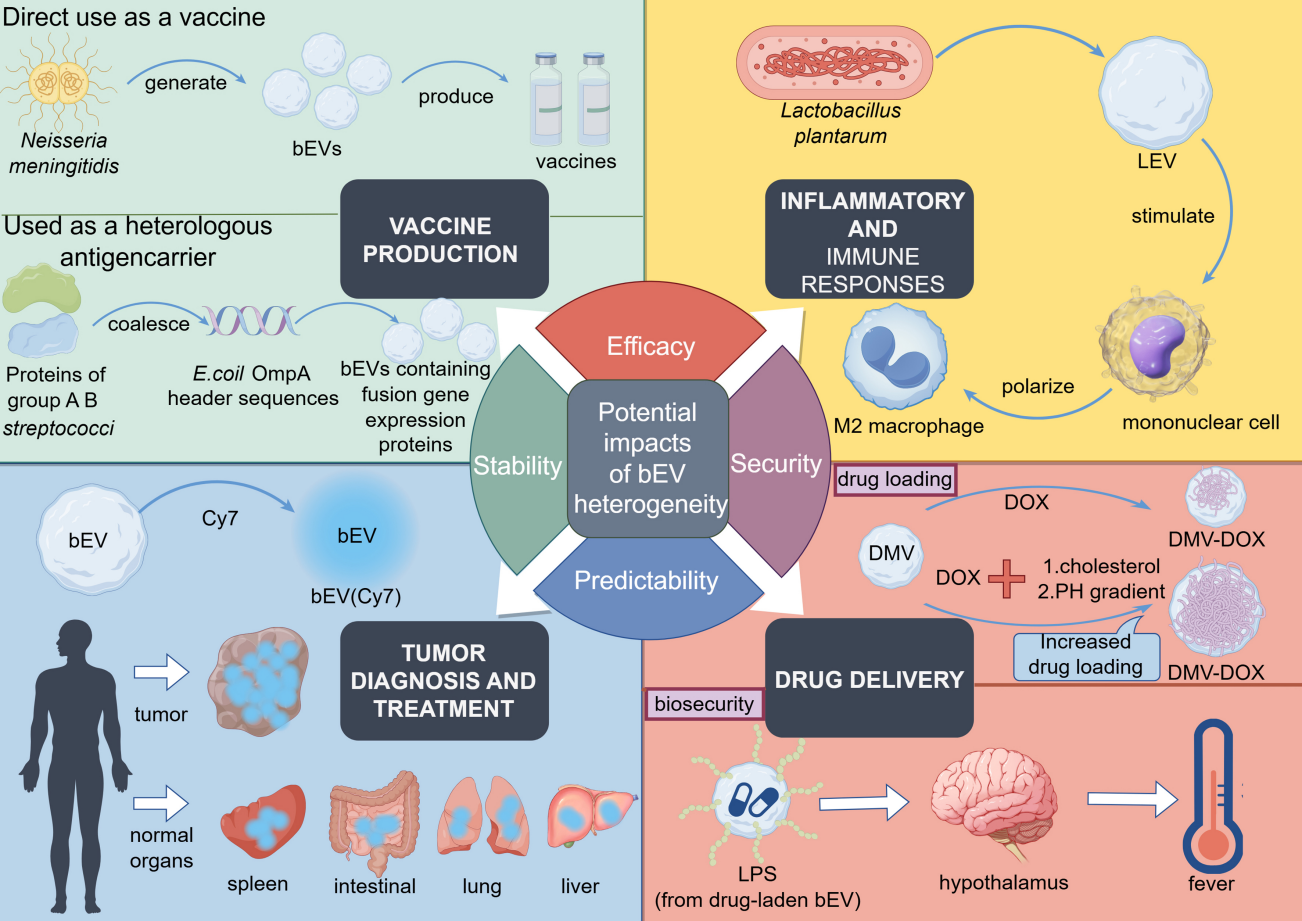
Examples of practical applications of BEVs and the potential impact of heterogeneity in their production and activities. (This figure was created using Figdraw). The figure shows examples of four application areas of BEVs and how their heterogeneity affects these applications. Vaccine manufacturing: An example is the direct use of BEVs derived from *Neisseria meningitidis* in vaccine production. Another example involves using BEVs as a vector for heterologous antigens. In this case, proteins from *group A and B streptococci* are fused with *E. coli* OmpA guide sequences, which are then expressed in the lumen of *E. coli*-derived BEVs, resulting in BEVs containing proteins from group A and B streptococci for use in vaccine production. Inflammation and immune response: The ability of LEV to promote the differentiation of monocytes into M2 macrophages in human THP-1 cells has been shown to play a role in anti-inflammation responses. Tumor imaging: BEVs were labeled with Cy7. *In vivo* fluorescence imaging showed that in addition to the high levels of BEV aggregation observed at the tumor site, different levels of BEV aggregation were also detected in the spleen, liver, heart, kidneys, lungs, and intestines. Drug delivery: The figure highlights two key aspects of drug delivery using BEVs - drug loading and biosafety. The drug loading section demonstrates that creating a pH gradient and adding cholesterol to drug-loaded vesicles can greatly improve the encapsulation efficiency of DOX. The biosafety section shows that LPS on the surface of BEVs can affect the human nervous system and cause heat generation, raising potential safety concerns in drug delivery applications. BEVs: Bacterial extracellular vesicles; LEV: BEVs derived from *Lactobacillus plantarum*; Cy7: a fluorescent dye; DOX: doxorubicin; LPS: lipopolysaccharides; DOX: doxorubicin; M2: macrophages: alternatively activated macrophages.

Heterogeneity in BEVs is a crucial factor affecting their effective application. Specifically, issues related to the purity, quantity, and reproducibility of BEVs remain major limitations in large-scale production, ultimately impacting subsequent in-depth research and broader applications of BEV components [Figure 1].

### Impact of BEV heterogeneity on vaccine production

BEVs are nanoscale membrane structures that facilitate intercellular communication and are involved in numerous physiological and pathological processes. Their relative accessibility and ease of manipulation make them promising candidates for development as carriers for vaccine antigens^[40]^. BEVs can be utilized in vaccines in two primary ways: either directly as vaccines themselves or as vectors for heterologous antigens.

The prevailing approach for using BEVs directly as vaccines aims to elicit a robust immune response without inducing cytotoxic effects. For example, there are currently four licensed BEV-based *Neisseria meningitidis* (*N. meningitidis*) vaccines from *N. meningitidis group B*, *N. meningitidis P1.7,16 strains*, and *N. meningitidis New Zealand 98/254 strain* of BEVs^[41]^. In addition, vesicles derived from non-pathogenic bacteria can also be engineered to express heterologous antigens from virulent pathogens. A key advantage of BEVs is their ability to be modified with specific proteins or polysaccharides from heterologous pathogens, enabling them to serve as effective antigen vectors. For instance, BEVs from *Escherichia coli* (*E. coli*) are often used as carriers for heterologous antigens. Proteins from *group A and group B Streptococcus* can be fused with *E. coli* OmpA guide sequences and expressed within the inner lumen of *E. coli*-derived BEVs. These modified BEVs have been shown to induce high levels of functional antibody titers in mice against the recombinant forms of these proteins^[42]^.

The use of BEVs as vaccines, whether directly or as vectors, is primarily challenged by the heterogeneity introduced during their production processes. As discussed above, various purification methods can significantly affect the uniformity of BEV preparations. Even when employing the same purification technique, variability among production batches persists. This process-related heterogeneity complicates the ability to produce large quantities of BEVs with the specificity and reproducibility required for effective vaccine formulations. To overcome this, it is crucial to develop standardized protocols for vesicle isolation and purification, as well as scalable production methods suitable for clinical applications^[43]^. Without addressing this issue, the inconsistencies can adversely affect the production, potency, and regulatory approval of BEV-based vaccines, thereby impeding their advancement in vaccine development.

### Impact of BEV heterogeneity on inflammatory and immune responses

BEVs are known to contain lipids, proteins, and RNA. Through mechanisms such as endocytosis, receptor-ligand interactions, or direct fusion with the cell membrane, BEVs can mediate and regulate various cellular processes in target cells, highlighting their significant role in inflammatory and immune responses *in vivo*^[40]^.

Macrophages, integral components of the mononuclear phagocyte system, are crucial for inflammation and immune regulation. They differentiate from monocytes that migrate into tissues and can polarize into two distinct active states influenced by microenvironmental signals: classically activated (M1) macrophages and alternatively activated (M2) macrophages. M1 macrophages dominate the early phase of inflammation, facilitating pathogen clearance and the recruitment of other effector cells. In contrast, M2 macrophages participate in anti-inflammation, wound healing, tissue remodeling, and angiogenesis during the resolution phase of inflammation. This balance between M1 and M2 macrophages is essential for maintaining tissue homeostasis. Recent studies have shown that BEVs derived from *L.plantarum* (LEV) preferentially promote the differentiation of monocytes into M2 macrophages in human THP-1 cells, effectively shifting the initial inflammatory M1 state toward the anti-inflammatory and tissue repair M2 state. This property of LEV presents promising potential for the treatment of inflammatory skin diseases^[44]^.

The above cases clearly demonstrate that BEVs represent a novel way to treat diseases. However, as an important tool for studying the immune regulation between pathogens and hosts, the consistency of BEVs must be carefully considered - both during pathogen infection and in investigations of related regulatory mechanisms. The small RNAs (sRNAs) of BEVs play an important role in the bacterial immune response to their hosts, but the heterogeneity of BEVs raises the question of whether they also affect the functions of host cells that internalize them. Among the thousands of unique sRNA sequences potentially carried by BEVs from *P. aeruginosa*, several are predicted to bind human mRNAs and thus influence the immune response. Nonetheless, due to the heterogeneity of BEVs, the occurrence of these few specific sRNAs is uncertain^[45]^. Another primary mechanism of BEV immune activation may involve the activation of toll-like receptors (TLRs) or other pathogen-recognizing receptors (PRRs), which recognize pathogen-associated molecular patterns (PAMPs) to promote an immune response^[46]^. Research has shown that the diverse cargoes carried by BEVs can activate different TLRs with different intensities and target distinct TLR receptors. This heterogeneity of BEVs makes it difficult to maintain a consistent immune response intensity when BEVs stimulate the host, posing challenges to ensuring their efficacy in immunization strategies^[47]^. Therefore, further studies are needed to assess how BEV heterogeneity affects the immune response, which is essential for optimizing their application in therapeutic and immunological contexts.

### Impact of BEV heterogeneity on drug delivery

BEVs are capable of transporting proteins, virulence factors, genetic materials, and lipids between different cells. This unique property has led to the emergence of BEVs as promising drug delivery systems. Compared to traditional drug delivery systems, BEVs offer more stable drug packaging, greater loading capacity, and enhanced biocompatibility. However, their heterogeneity poses several challenges that must be addressed.

The primary challenge for the use of BEVs in pharmaceutical clinical practice is biosafety. The presence of LPS on their surfaces, along with the toxic factors they produce, poses significant risk factors^[48]^. LPS, which consists of lipid A, core polysaccharide, and the O-specific antigen chain, can lead to febrile reactions and endotoxic shock. Notably, LPS-deficient EVs (nEVs) exhibit lower immunogenicity compared to BEVs with normal LPS levels, thereby improving safety at the expense of potency and compromising the vaccine’s ability to elicit cross-protective immunity^[49]^. In addition to LPS, virulence factors released by the parental bacteria can also pose toxicity risks. For instance, BEVs derived from *Pseudomonas aeruginosa* (*P. aeruginosa*) deliver various virulence factors, including Cif, alkaline phosphatase, β-lactamase, and hemolytic phospholipase C, into the cytoplasm of airway epithelial cells. These various virulence factors promote biofilm formation, degrade antimicrobial peptides, and reduce ciliary clearance^[50]^. Therefore, the heterogeneity of BEVs can lead to inconsistent content of these various virulence factors in their packaging, which can affect their safety as drug delivery systems.

Furthermore, the membrane composition, pH, and osmolarity of BEVs can significantly influence their chemical stability and permeability, ultimately impacting drug encapsulation and release. Higher permeability in BEVs can lead to easier drug release, thereby increasing drug loading capacity. Some researchers have demonstrated that generating a pH gradient and incorporating cholesterol into the drug-loaded vesicles can substantially enhance the efficiency of doxorubicin (DOX) encapsulation. Specifically, this approach has reportedly increased drug loading to 12% (w/w) and saturated the efficiency with the addition of 10% cholesterol^[51]^. Therefore, the variability in BEV permeability plays a crucial role in drug loading and release dynamics.

### Impact of BEV heterogeneity on tumor diagnosis and treatment

BEVs possess good stability, permeability, immunogenicity, and tumor-targeting properties, making them widely applicable in tumor diagnosis and therapy. However, BEVs are influenced by various factors that lead to variations in their content and external properties. This heterogeneity specifically impacts their effectiveness in tumor diagnosis and treatment.

BEVs can serve as carriers for antitumor drugs, enabling targeted delivery and enhanced penetration into tumor lesions. For instance, DOX-BEVs - comprising BEVs produced by attenuated *Klebsiella pneumoniae* (*K. pneumoniae*) and the broad-spectrum antitumor agent DOX - have demonstrated potent tumor growth inhibitory effects. Studies have shown that DOX-EVs accumulate in NSCLC A549 cells, enabling their use for the treatment of non-small cell lung cancer^[52]^. Moreover, BEVs derived from *Salmonella typhimurium* can stimulate specialized antigen-presenting cells and enhance the production of proinflammatory factors such as NO, TNF-α, and IL-12, which can synergize the antitumor effect due to the LPS and virulence factors they carry from the parental bacteria^[53]^. Thus, heterogeneity in BEVs may lead to differences not only in drug delivery *in vivo* but also in the immune response, ultimately affecting the overall therapeutic efficacy.

Beyond that, it is worth noting that there are still considerable risks associated with using natural BEVs to treat tumors. Their heterogeneity may contribute to off-target effects, causing BEVs to accumulate in unintended host organs. For instance, *in vivo* fluorescence imaging of Cy7-labeled BEVs following intravenous injection revealed that although high levels of BEV aggregation were detected at tumor sites, varying levels of BEV aggregation were also detected in the spleen, liver, heart, kidney, lung, and intestines. This off-target accumulation suggests potential risks to normal tissues and organs, posing safety concerns^[54]^.

## FACTORS AFFECTING CONSISTENCY IN BEV PREPARATION AND PROSPECTS FOR MODIFICATIONS

**Figure 2:**
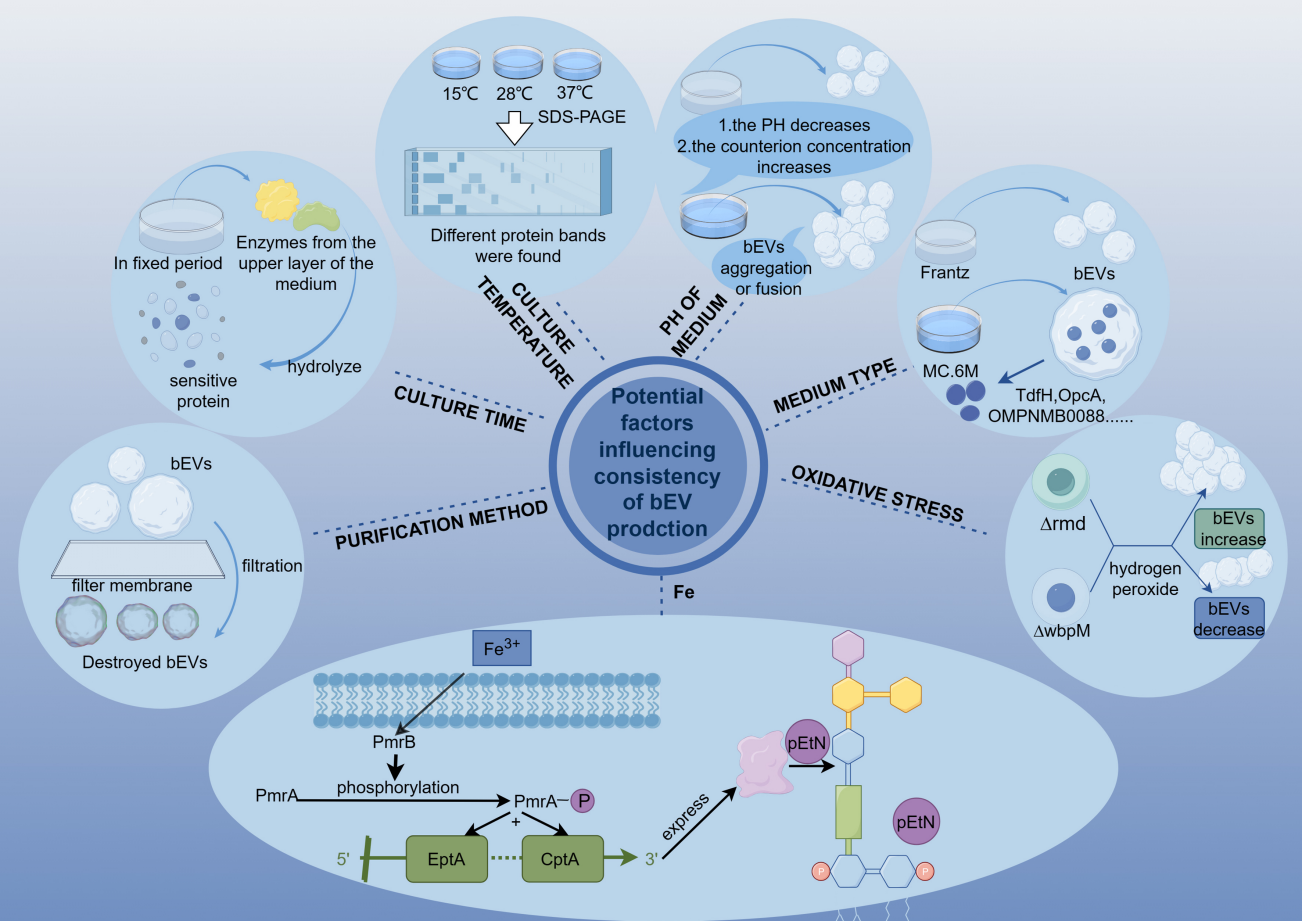
Examples of factors that can influence various characteristics of BEVs (This figure was created using Figdraw). This figure illustrates seven potential factors that affect the consistency of BEV production. Effect of purification method: The figure demonstrates the disruption of BEV structure caused by filtration through a membrane, emphasizing how the choice of purification method influences the integrity of the vesicles. Culture time: It illustrates that extracellular proteases secreted during the stationary phase break down certain proteins t sensitive to these enzymes. Variations in the length of the stationary phase lead to inconsistencies in the degradation levels of these proteins, leading to differences in the protein profiles of isolated BEVs. Temperature influence: The figure illustrates the BEVs obtained from bacteria cultured at 15, 28, and 37 °C. SDS-PAGE analysis reveals differences in the protein composition and content of BEVs under these conditions, highlighting that temperature affects BEV consistency. Effect of pH: It shows that acidic pH and/or high counterion concentrations induce aggregation or fusion of BEVs, reflecting the effect of pH on BEVs. Role of culture medium: The figure depicts that BEVs produced in MC.6M medium contain more specific proteins such as TdfH, OpcA, OMP NMB0088, *etc*., compared to BEVs from bacteria cultured in Frantz medium. This underscores the influence of the culture medium on BEV consistency. Iron regulation: This figure illustrates how iron activates the PmrAB system. Iron first activates PmrB, which then phosphorylates PmrA. This activation regulates the addition of ethanolamine phosphate to lipid A and the LPS core by EptA and CptA, demonstrating iron’s effect on BEV lipid composition. Oxidative stress and vesicle production: The figure shows that hydrogen peroxide-induced BEV generation requires the presence of Δ*wbpM* S, but not Δ*rmd*. This reflects that oxidative stress can be strategically used to produce vesicles. BEVs: Bacterial extracellular vesicles; TdfH, OpcA, OMP NMB0088: proteins expressed in BEVs; Δ*wbpM*: A B-band LPS deletion mutant; Δ*rmd*: an A-band LPS deletion mutant; LPS: lipopolysaccharides.

As an emerging biotechnology tool, BEVs are believed to have a wide range of potential applications. However, the expanding applications of BEVs are significantly affected by heterogeneity. To address the heterogeneity issues that may arise during the application of BEVs, it is crucial to deeply understand and explore the influencing factors and the possible mechanisms. By addressing these problems during the generation and manufacture of BEVs, their utilization can be effectively improved, and their application value in medicine and biotechnology can be enhanced. Several common factors that contribute to heterogeneity in BEVs are summarized below [Figure 2].

### Purification methods

As discussed previously, numerous purification techniques are available for isolating BEVs. Differences in the purification methods employed can result in significant heterogeneity in the quantity, quality, and functionality of the obtained BEVs, even between batches produced using the same technique. BEVs isolated via sucrose density gradient exhibited greater heterogeneity in size and density, and failed to clearly segregate subpopulations of BEVs with distinct sizes, such as exosomes and microvesicles, compared to iodide density gradient^[55]^. Ultracentrifugation is regarded as the most extensively utilized method for obtaining BEVs, owing to its low cost and capability to isolate BEVs from large volumes of biofluids. However, the centrifugation processes may pellet non-exosome contaminants of similar densities, which may affect the consistency of the isolated BEVs. Additionally, the forces exerted during ultrafiltration to pass the sample through the membrane may result in damage or deformation of the BEVs, thus further impacting the consistency of the isolated BEVs^[56]^.

To utilize BEVs as vaccine agents and drug delivery vehicles, large quantities of high-quality BEVs must be prepared. The current challenges in implementing separation and purification methods using ultracentrifugation at an industrial scale suggest the need to optimize purification methods capable of handling large quantities of BEVs. Furthermore, the establishment of assessment methods and concepts that rigorously define BEV quality, including quantity, purity, particle size, distribution, homogeneity, and potency, is also necessary^[57]^. Depending on the characteristics of the BEVs and the downstream application, a combination of different techniques may offer the possibility to efficiently obtain BEVs of high purity and yield. For example, low-speed differential centrifugation combined with microfiltration for pre-cleaning can be used to remove biological fluids, cell culture supernatants, cells, cell debris, and other large particles. Subsequently, a 30%-60% sucrose buffer pad or iodixanol can be employed in the ultracentrifugation step to remove vesicle-free proteins or protein-RNA aggregates, thereby enhancing the purity of the BEVs^[58]^. Currently, the different components are still identified using density gradient centrifugation. In this way, it is possible to know which density gradient has the highest content and purity of BEVs and, thus, to determine the most suitable gradient for the separation of BEVs. Therefore, finding a simple visualization method to quickly identify the most suitable gradient for BEVs is a future endeavor. In addition to the above-mentioned traditional methods such as filtration, centrifugation, and density gradients, many new techniques have been used to purify vesicles. Microfluidics is an emerging separation method that relies on a series of sEV characteristics, such as immunoaffinity, density, and size, to overcome the limitations of traditional methods. This method offers advantages including low cost, small size, high speed, high sensitivity, no labeling, and high recovery, and is expected to replace traditional methods, playing a crucial role in the industrialization and mass production of BEVs in the future^[59]^. In addition, purification methods can lead to large variations and inconsistencies in the dosage of BEVs during preclinical and clinical studies, which highlights the limitations of quantitative methods during production, and therefore, there is an urgent need to establish consistency in the quantification of EVs. Nanoparticle tracking analysis (NTA) is an invaluable tool for the characterization of EVs, providing insights into EV size, size distributions, concentrations, and heterogeneity. It can therefore be combined with purification methods to optimize the production of BEVs^[60]^.

### Cultivation time

Bacteria cultured in liquid medium undergo both an exponential growth phase and a subsequent stationary phase. *Francisella novicida* (*F. novicida*) is observed to produce a distinct profile of BEVs-associated proteins during the exponential growth phase, as compared to the stationary phase. The amount of proteins in BEVs derived from *F. novicida* during the stationary phase is approximately three times higher than that observed in the exponential phase, and the specific protein species present also differ. This trend was not limited to *F. novicida*, as a similar observation was made for *P. aeruginosa* through SDS-PAGE analysis, which revealed differences in the protein content, protein species, surface electron charge, and *Pseudomonas* quinolone signal (PQS) content of BEVs collected at different time points. The membrane vesicles of *P. aeruginosa* are known to contain a quorum sensing (QS) signaling molecule, 2-heptyl-3-hydroxy-4-quinolone (PQS). PQS is a mediator of intercellular communication and is transported in the vesicles of this organism. It regulates bacterial gene expression to influence bacterial physiological functions, pathogenicity, and biofilm formation. It has been shown that the accumulation of negative charges caused by the addition of PQS to LPS creates repulsion, which generates forces sufficient to bend the membrane, ultimately leading to vesicle outgrowth. Thus, PQS also plays an important role in stimulating vesicle production, which is one of the key regulators of vesicle production in *P. aeruginosa.* For example, PQS biosynthesis mutants produce significantly fewer BEVs. Furthermore, the addition of exogenous PQS was shown to restore the production of BEVs in mutants lacking the PQS receptor as well as in PQS-deficient cells whose protein synthesis was inhibited by antibiotic treatment^[61,62]^. The authors hypothesize that culture time affects the consistency of BEVs through two primary mechanisms: first, it is related to the presence of extracellular proteases in the upper layer of the culture medium, as many proteases are secreted during the stationary phase, which can affect the catabolism of certain protease-sensitive proteins. When the length of the stationary phase varies, the degree of catabolism of these protease-sensitive proteins becomes inconsistent, thus leading to differences in the protein profiles of the isolated BEVs. Secondly, bacteria may employ different mechanisms to produce BEVs during various growth phases. For instance, in *P. aeruginosa*, the electronegative charge of LPS leads to charge repulsion between neighboring LPS molecules, which can facilitate outer membrane blistering and BEV release. Notably, PQS enhances this charge repulsion, further strengthening the outer membrane blistering process and BEV production. During the stationary phase, *P. aeruginosa* relies more heavily on this charge-driven blistering mechanism to generate BEVs. In contrast, during the exponential growth phase, *P. aeruginosa* utilizes an alternative BEV biogenesis pathway that does not depend on PQS, resulting in differences in the electronic charge and PQS content of the BEVs produced in the different growth phases, ultimately affecting the overall consistency of the isolated BEVs^[63]^.

BEVs should be collected from cells at the same stage of growth to improve the consistency of the samples. Grouping BEVs collected at different growth stages into the same batch is not recommended. For instance, the BEV production of *Capnocytophaga ochracea* is observed to be higher during the logarithmic growth phase and significantly lower during the stationary phase. This finding suggests that the logarithmic growth phase can be leveraged to harvest a larger quantity of BEVs. Additionally, the size distribution of BEVs is observed to be dependent on the growth stage. BEVs produced during the logarithmic growth phase tend to have larger aggregates, whereas those from the stationary phase are predominantly within the 20-150 nm range. These observations indicate that the choice of growth stage for BEV harvesting should be tailored to the specific application requirements^[64]^.

### Temperature of the medium

The appropriate temperature is crucial for the survival of bacteria within the medium. Fluctuations in the medium temperature can affect the content of BEVs, including proteins and LPS, thereby influencing the consistency of BEVs. Analysis of BEVs from *Yersinia enterocolitica* using the BCA assay revealed that the protein content of BEVs was higher at 28 °C compared with 37 °C. Furthermore, SDS-PAGE analysis revealed a unique protein present in BEVs cultured at 15 °C. Additionally, a prominent 50 kDa protein was observed in BEVs cultured at 28 °C, whereas its intensity was lower in BEVs cultured at 15 and 37 °C, suggesting that the 50 kDa protein was most abundant in BEVs cultured at 28 °C. What proteins are altered needs to be determined by subsequent proteomics analysis. The findings presented above suggest that temperature influences the composition and content of proteins in BEVs from *Yersinia enterocolitica* (*Y. enterocolitica*), thereby affecting the consistency of the BEVs^[65]^.

When collecting BEVs, it is important to first determine whether the temperature has any effect on their production. If so, the temperature of the culture medium should be controlled within a narrow range to maintain the consistency of the BEV samples. For instance, under *in vitro* conditions, the bacterium *Serratia marcescens* is observed to produce a large number of vesicles at 22 or 30 °C, but a negligible number at 37 °C^[66]^.

### pH of the culture medium

BEVs exhibit varied stability under different pH conditions. Dynamic light scattering (DLS) is a commonly used technique to probe the stability of suspended vesicles. DLS measures the average particle size of the vesicles and calculates the aggregation kinetics by measuring the change in particle size. DLS measurements have demonstrated that suspensions of BEVs derived from native *E. coli* (Nissle 1917) and LPS-modified *E. coli* (ClearColi) strains exhibit aggregation and/or vesicle fusion at acidic pH values exceeding the experimentally determined isoelectric point, as well as at high counterion concentrations above the critical coagulation concentration (CCC)^[67]^. Furthermore, it has been reported that alterations in environmental pH also influence the protein composition of BEVs. *Vibrio fischeri* modulates the levels of its primary outer membrane protein, OmpU, in order to adapt to the acidic pH conditions experienced during the transition from the environment to the host. Studies have shown that BEVs with elevated OmpU levels under acidic pH conditions are capable of more effectively stimulating the development of the symbiotic host with the bacteria^[68]^. In a recent study, it was found that the lipid content of *Lactobacillus rhamnosus* (*L. rhamnosus)* extracellular vesicles (LREVs) varies with the pH of the environment, and that the lipid content likewise varies with the incubation time and the nutrient composition of the medium^[39]^. Lipid changes are one aspect that potentially affects vesicle consistency and can have important downstream effects on vesicle cargo composition. Taking PLs as an example, in a recent study of novel mechanisms of OMV biogenesis in Gram-negative bacteria, it was noted that when PLs accumulate in the outer membrane leaflets, this leads to an increased release of BEVs, which are enriched in PLs and certain fatty acids compared to BEVs released in the normal state of PL accumulation^[69]^.

The pH of the solution should be regularly monitored during BEV production to ensure it remains within the appropriate range, avoiding any adverse effects on the vesicles. If necessary, suitable pH adjusters may be utilized to modify the solution pH during production for specific purposes. For example, aggregation and fusion of BEVs can be induced by adjusting pH and salt concentration. Acidic pH and high salt concentration can promote the aggregation of BEVs, while low pH can induce the fusion of BEVs. This information can provide guidance for the preparation of BEV-based vaccines or therapeutic formulations containing diverse antigens^[67]^. Different bacteria may produce BEVs most efficiently at different pH. In studying the optimum pH for BEV production by *Francisella tularensis* between pH 7.2 and 4.8, it was found that the highest production of BEVs occurred at pH 5.3^[14]^. It has been noted that a pH of 3.5 induced the highest LREV production; however, studies on *Helicobacter pylori* have shown that low pH values reduce the production of their BEVs^[39]^.

### Type of culture medium

The type of growth medium utilized can significantly impact the production of BEVs, including influencing the abundance and distribution of BEV-associated proteins. A comparative study of the BEVs derived from *N. meningitidis* cultured in two distinct media, Frantz and MC.6M, revealed differences not only in their protein content but also in their immunogenicity. For instance, certain differentially expressed proteins, such as TdfH, OpcA, OMP NMB0088, hypothetical NMB2134, lipoprotein NMB1126/1164, and NspA, were found to be significantly upregulated in the BEVs produced by bacteria cultured in the MC.6M medium^[70]^. These findings suggest that variations in the growth media utilized to produce BEVs-based vaccines may influence antigen expression and protein composition, thereby ultimately affecting the consistency of the final product. In the study of *Haemophilus parahaemolyticus* for BEV applications, determining the appropriate growth conditions for the bacteria is the beginning of the process, as the medium and culture method have a great influence on the protein content and yield of BEVs^[71]^. Similarly, in the study on *L. rhamnosus*, it was mentioned that different broth concentrations in the culture medium affect the content of proteins and lipids in LREVs^[39]^.

When researching and producing BEVs, the effects of the culture medium type on experimental results must be considered. A suitable culture medium should be selected, and this factor should be carefully controlled during experimental design and data analysis. Additionally, attention should be given to even seemingly trivial factors, such as batch-to-batch variations in media from the same manufacturer.

### Iron in the medium

Iron is a crucial component in bacterial culture media and has been demonstrated to significantly influence the production and composition of BEVs. Studies have shown that iron deficiency in the medium affects the expression of virulence factors associated with BEVs in *Helicobacter pylori* (*H. pylori*), leading to alterations in the LPS levels and the composition of the BEV envelope^[72]^. Additionally, a separate study found that the protein and lipid membrane composition, size, and elasticity of BEVs released from bacteria cultured under iron-deficient or starvation conditions differed significantly compared to the control BEVs^[73]^. LPS is an important component of the outer membrane and acts as a permeability barrier. Covalent modifications of LPS have been shown to affect the biogenesis of BEVs. Several intestinal bacteria utilize iron-regulated enzymes, such as EptA and CptA phosphoethanolamine transferases, to modify their LPS. The two-component system PmrAB senses and adapts to environmental iron levels, regulating the transcription of EptA and CptA. Activation of the PmrAB system by trivalent iron and mild acidity modulates the addition of ethanolamine phosphate to the lipid A and LPS core fractions by EptA and CptA, respectively. Studies have demonstrated that the protein and lipid concentrations of BEV preparations from strains lacking the EptA and CptA enzymes were approximately 2-fold higher at the same iron concentration, suggesting that these genes have a negative impact on BEV formation and alter the composition of the BEVs^[74]^.

Continuous monitoring of the iron concentration in the culture medium is particularly critical. The iron concentration can be measured using a colorimetric method that employs a specific indicator, such as chrome azurol S, which reacts with iron ions to produce a color change. The iron concentration is then estimated by comparing the color depth, thereby improving the consistency of the BEVs produced. Under iron-limiting conditions *in vivo*, the VacJ/Yrb ABC transporter system is downregulated, which has been found to result in increased BEV production in *Vibrio cholerae* and *Haemophilus influenzae*. This regulatory mechanism is highly conserved among Gram-negative bacteria, thus providing a means of regulation^[69]^. It is worth noting that varying concentrations of iron have different effects on distinct species of bacteria, making it challenging to measure these effects using a uniform standard. For example, Olczak *et al*. experimentally demonstrated that when *Porphyromonas gingivalis* was grown under low iron/heme conditions and supplemented with iron chelators, the production of the protein HmuY increased significantly. This protein was more abundant in the OMV fraction. However, the production of this protein was low in bacteria grown under high iron/heme conditions^[75]^. In contrast, Andrew Gorringe *et al*. showed that no significant differences in immunoprotection were observed between iron-restricted and iron-enriched cultures during the development of a vaccine against meningococcal disease based on *Neisseria lactamica* OMVs^[76]^. The above examples illustrate that the effects of varying iron concentrations in culture are strain-specific. For some bacterial strains, iron sensitivity influences the production or nature of certain substances. In contrast, other strains are not sensitive to iron, with no discernible differences observed between low and high iron levels. Thus, the practical application of modulating iron concentration to influence BEVs should consider strain-specific characteristics.

### Oxidative stress in culture

Bacteria may release vesicles as a defense mechanism in response to oxidative stress. Treatment with peroxides mimics the burst of reactive oxygen species produced by neutrophils in response to bacteria during infection, thereby increasing BEV production. For instance, an investigation of the response of *P. aeruginosa* to oxidative stress has shown that D-cycloserine treatment resulted in a 9.2-fold increase in BEV production. The presence of B-band LPS is closely related to this phenomenon^[62]^. A prominent virulence factor of *P. aeruginosa* is LPS, an immunodominant antigen located on the outside of the outer membrane, which produces two O antigens attached to the lipid A+ core: the B-band O antigen and the A-band O polysaccharide. The B-band O antigenic repeating unit of the LPS is responsible for serotype specificity; strains lacking the O antigen have been shown to be less virulent in animal models of infection^[77]^. The A-band O polysaccharide holds considerable promise as a vaccine or therapeutic target^[78]^. Consistent with rejection-mediated membrane bending effects, native OMVs have also been found to be highly enriched in LPS with the longer and highly charged form of the O antigen (B-band LPS), in contrast to *P. aeruginosa* outer membranes that contain the short, uncharged A-band and B-band LPS^[62]^. Studies on *P. aeruginosa* by Ian A Macdonald *et al*. have shown that peroxide-induced OMV production requires the presence of B-band but not A-band LPS. For example, Δ*wbpM*, a B-band LPS deletion mutant, showed a 60% reduction in BEV production by hydrogen peroxide treatment. Δ*rmd*, an A-band LPS deletion mutant, showed an approximately 7-fold increase in BEV production by hydrogen peroxide stress^[62]^.

The judicious application of oxidative stress can stimulate BEV production without compromising the vesicle components and properties, thereby maintaining consistency. This approach aims to enhance production efficiency as much as possible. For example, in a study on *N. meningitidis*, cysteine depletion was found to induce oxidative stress, which is an intracellular signal that increases vesicle formation. The study suggests that this approach may be applicable to vaccine production^[79]^.

## CONCLUSIONS AND PROSPECTS

### Limitations

Although BEVs show great potential, their research and application in medical sciences still face many significant challenges. Among them, the isolation and purification of BEVs are particularly important. Only by obtaining high-quality and consistent vesicles through appropriate methods can the accuracy of subsequent analyses and diagnostic treatments be ensured. Currently, the separation of BEVs relies mainly on ultracentrifugation, a method that raises concerns about its time-consuming and energy-consuming nature^[80]^. Existing techniques make it difficult to achieve a complete separation of BEVs from the parent bacterial lysis products. To overcome these challenges, researchers have explored combinations of purification methods, such as ultrafiltration, density gradient centrifugation, and immunoaffinity. In addition, the application of emerging technologies, such as microfluidics^[27]^ and NTA^[60]^, has made significant advances in the purification process. Therefore, future research should focus on the improvement of these techniques to improve the consistency of BEVs, thus providing a more reliable basis for relevant studies. In addition, it is worth noting that the natural PAMP and immunostimulatory antigens present in BEV, while enabling BEV to exhibit unique immunogenicity and serve as a promising vaccine platform, also play an important intrinsic role in bacterial pathogenicity. Therefore, the balance between biosafety and immunostimulatory properties of BEV-based vaccines should be carefully considered. When the consistency of a BEV is difficult to ensure, and the amount of virulence factors (e.g., virulence proteins and LPS) in a BEV cannot be determined, excessive immune stimulation, inflammatory response, reactogenicity, or other adverse reactions may result. Going forward, additional measures need to be refined to improve the consistency of BEVs and to accurately control the concentration of harmful bacterial components of BEVs that pose a threat to the host, thereby minimizing adverse effects^[81,82]^.

### Conclusions

Practical applications of BEVs have become a research focus since their discovery, owing to their unique nanoscale structure, as well as their favorable encapsulation, targeting, and biocompatibility properties. They exhibit an extremely broad application prospect. This paper reviews the structural composition and biological functions of BEVs. A variety of mainstream methods are summarized herein to help mitigate potential unintended negative impacts that can result from undesirable physical characteristics and composition of BEVs. Attention to this information should help facilitate a range of BEV applications. However, BEV production is readily influenced by the external environment. This is reflected in variations in purity and quantity, which restricts their large-scale production and extended application. Therefore, this review provides an overview of the connotations of BEV heterogeneity and a comprehensive explanation of the specific limitations that this discrepancy imposes on vaccine production, oncology treatments, and other applications, such as wound healing, lung inflammation, burn care, periodontal disease, etc. Additionally, this paper explores the influence of various factors on BEV contents and yield, which provides a more systematic framework for subsequent related research. Documenting factors that influence the consistency of BEV preparations among researchers, as well as those that impact standard operating procedures of large-scale production for therapeutic applications, is expected to enable breakthroughs and progress in the field of human health and medical treatment.

## DECLARATIONS

### Copyright

© The Author(s) 2025.
